# The Rewards of Science

**Published:** 1874-04

**Authors:** 


The Rewards of Science.—Whoever, in the pursuit of science, seeks after immediate practical utility, may generally rest assured that he will seek in vain. Each individual student must be content to find his reward in rejoicing over new discoveries, as over new victories of mind over reluctant matter, or in enjoying the æsthetic beauty of a well ordered field of knowledge, where the connection and the filiation of every detail is clear to the mind and whose all denotes the presence of a ruling intellect; he must rest satisfied with the consciousness that he too has contributed something to the increasing fund of knowledge on which the dominion of man over all the forces hostile to intelligence reposes. He will indeed not always be permitted to expect from his fellow-men, appreciation and reward adequate to the value of his work. It is only too true that many a man to whom a monument has been erected after his death, would have been delighted to receive during his lifetime a tenth part of the money spent in doing honor to his memory. At the same time we must acknowledge that the value of scientific discoveries is now far more fully recognized than formerly by public opinion, and that instances of authors of great advances in science, starving in obscurity have become rarer and rarer. Helmholtz.

